# Comparative Evaluation of the Effect of Six Different Low-Surface-Tension Vehicles on the Penetration of Modified Triple Antibiotic Paste in Dentinal Tubules: An In Vitro Study

**DOI:** 10.7759/cureus.44939

**Published:** 2023-09-09

**Authors:** Aradhana N Kibe, Pradnya P Nikhade, Akshayprasad P Thote

**Affiliations:** 1 Conservative Dentistry and Endodontics, Bharati Vidyapeeth (Deemed to be University) Dental College and Hospital, Sangli, IND; 2 Dentistry, Sharad Pawar Dental College, Datta Meghe Institute of Medical Science, Wardha, IND; 3 Conservative Dentistry and Endodontics, Prakash Institute of Medical Sciences & Research, Islampur, IND

**Keywords:** sodium ether lauryl sulfate, confocal laser scanning microsopy, polyethylene glycol, chitosan, chlorhexidine gluconate, neosporin h, bupivacaine, modified triple antibiotic paste

## Abstract

Background

Modified triple antibiotic paste (MTAP) helps in the elimination of microorganisms which is imperative for the success of endodontic therapy. The intracanal medicament powders must be mixed with vehicles for better handling and penetration in the root canals. The purpose of this study is to compare the effect of six low-surface-tension vehicles on the penetration of MTAP in dentinal tubules.

Methodology

The root apices of 60 single-rooted human mandibular premolars were resected to obtain 12mm length. After biomechanical preparation, intracanal medicaments were prepared by mixing with the six vehicles (Group 1- Bupivacaine, Group 2- Sodium ether lauryl sulfate, Group 3- Neosporin H, Group 4- Chlorhexidine gluconate, Group 5- Chitosan, and Group 6- Polyethylene glycol) with MTAP and Rhodamine B dye. Middle and apical transverse sections were scanned under a confocal laser scanning microscope. The data were statistically analyzed using the one-way ANOVA test and the level of significance was p<0.05.

Results

The maximum depth of penetration was seen in Group 2 (MTAP with sodium ether lauryl sulfate) followed by Group 5 (MTAP with chitosan), Group 4 (MTAP with chlorhexidine gluconate), Group 1 (MTAP with bupivacaine), and Group 6 (MTAP with polyethylene glycol), and the least penetration by Group 3 (MTAP with Neosporin H). The depth of penetration in the middle level was in the order of Group 2 followed by Group 4, Group 5, Group 3, Group 6, and Group 1. The depth of penetration in the apical level was in the order of Group 2 followed by Group 5, Group 1, Group 4, Group 6, and Group 3. The overall depth of penetration was significantly higher at the middle level than at the apical level.

Conclusion

Group sodium ether lauryl sulfate showed the maximum depth of penetration in both the middle and apical areas. The least depth of penetration in the middle area was seen in group bupivacaine and the apical area by group Neosporin H

## Introduction

For the triumph of endodontic therapy, the elimination of microbial flora like Enterococcus faecalis, Prevotella intermedia, Eubacterium, Porphyromonas endodontalis, etc. from the root canals and fins is imperative [1,2]. Due to the inability of systemic medications to reach the site of infection in greater quantities, local drug transfer is preferred. The intra-canal medicament is a drug that is placed in between clinical appointments in the root canals [3]. The various medications are calcium hydroxide, poly antibiotic paste, double antibiotic paste (DAP), triple antibiotic paste (TAP), modified triple antibiotic paste (MTAP), etc. The benefit is the elimination of microorganisms and the induction of healing. These medications are available in powder form. So, for proper handling and introduction into the root canal, low-surface-tension vehicles are used. It is a known fact that capillary penetration increases with low surface tension [4]. Hence, this study aimed to compare the effect of six low-surface-tension vehicles on the penetration of MTAP in dentinal tubules.

## Materials and methods

**Figure 1:**
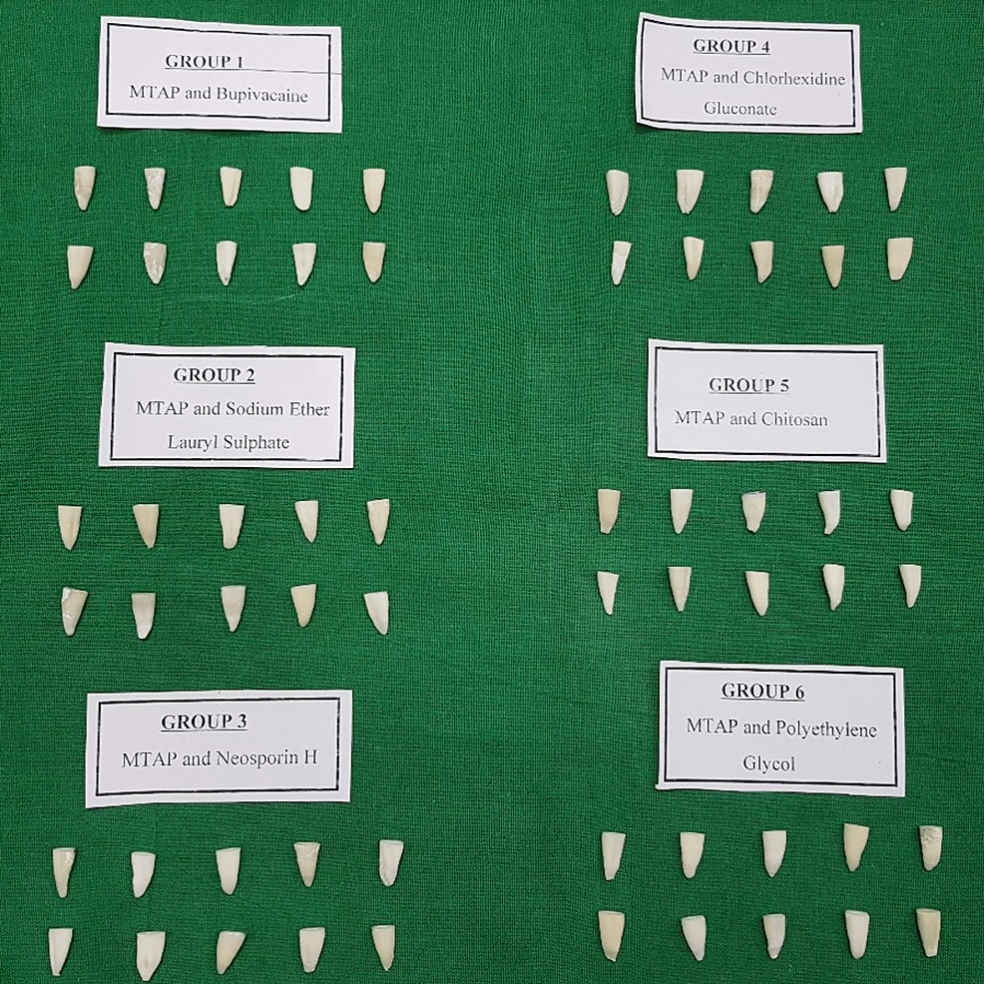
Sixty decoronated single-rooted premolars randomly divided into six groups: Group 1- MTAP and bupivacaine, Group 2- MTAP and sodium ether lauryl sulfate, Group 3-MTAP and Neosporin H, Group 4- MTAP and chlorhexidine gluconate, Group 5- MTAP and chitosan, and Group 6- MTAP and polyethylene glycol

The study was conducted in the Department of Conservative Dentistry and Endodontics, Sharad Pawar Dental College and Hospital, Sawangi, India. Sixty single-rooted human mandibular premolars with mature apices that were extracted for orthodontic purposes were selected and stored in a humidifier in a sterile environment until the experiment. The crowns of the teeth were to obtain standardized lengths of 12 mm. Canals were located, and the working length was determined using the hand K files (Mani, India). Biomechanical preparation of all the roots was done using rotary tapers up to F2 tapers using an Xsmart Endomotor (Dentsply, India). The root canals were irrigated with 5 ml of 17% EDTA (Prevest Dent Pro India), 10 ml of 3% sodium hypochlorite (Vishal Dentocare PVT. Ltd.), and 5 ml of distilled water. Paper points were used to dry the root canals. The 60 samples were randomly assigned to six groups (n = 10), as shown in Figure 1.

**Figure 2:**
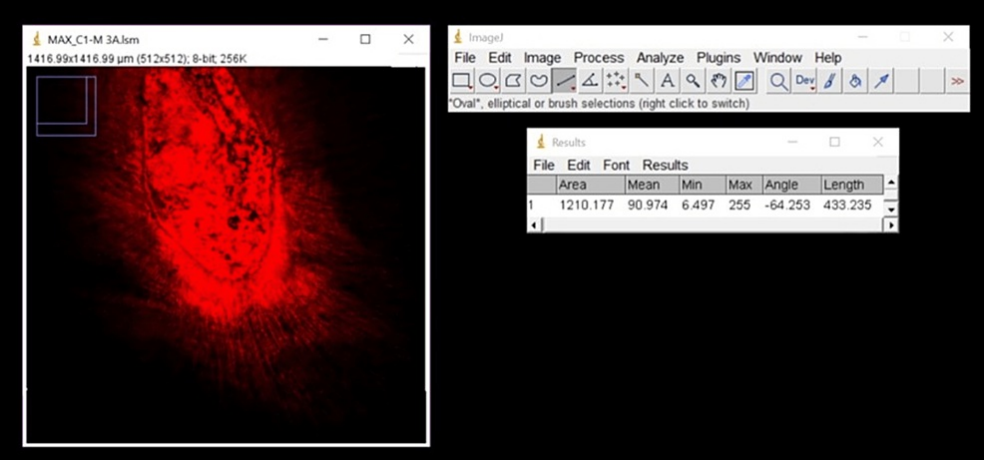
Evaluation of confocal laser scanning microscopic images using Image J software

MTAP was prepared by grinding the tablets of ciprofloxacin (Cipla), clindamycin (Cipla), and metronidazole (JB Chemicals) in 1:1:1 by weight in a mortar and pestle. 0.1% Rhodamine B dye was added to all the groups. The mixtures were transported to the root canals, and the depth of penetration was improved using No. 25 Lentulospiral (Mani) at 900 rpm. The samples were sealed with the temporary material (T-fill, Maarc, India). All 60 specimens were incubated in an incubator at room temperature for one day. The 1 mm-thick slices were obtained using diamond disks angled 90° to the long axis of each root at the middle and apical thirds. The samples were scanned under a Zeiss LSM 780 Laser Scanning Confocal Microscope (Carl Zeiss, Germany) under 5x magnification. The images were assessed via Image J software (NIH) (Figure 2). For each sample, "the depth of penetration was the deepest point of penetration measured from the canal wall." The data were statistically analyzed using a one-way ANOVA test, and the level of significance was p <0.05.

## Results

Depth of penetration of six groups in the middle level of the root

**Figure 3:**
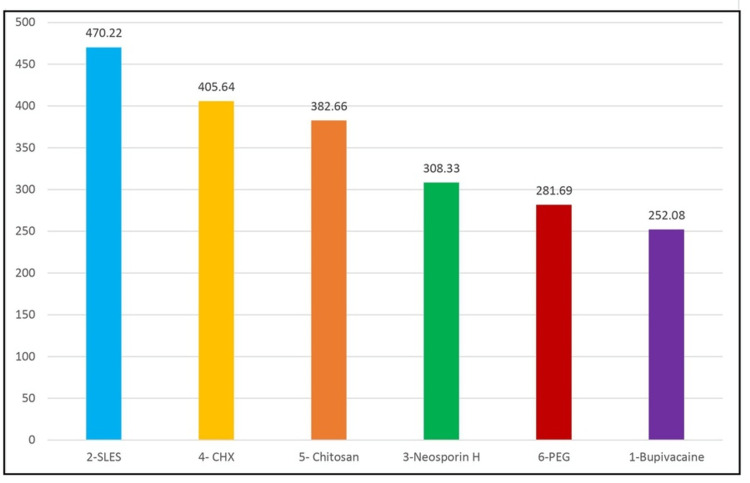
Comparison of the depth of penetration into dentinal tubules at the middle level of six groups (µm)

The mean depth of vehicle penetration in the middle level of the root for Group 1 was 252.0813 ±11.06681 µm, that for Group 2 was 470.2221 ± 25.32511µm, that for Group 3 was 308.3281±8.976426 µm, that for Group 4 was 405.6382 ± 14.64477 µm, that for Group 5 was 382.6633± 11.71669 µm, and that for Group 6 was 281.6891± 11.86847 µm (Table 1). The order of depth of penetration in the middle level as shown in Figure 3 is Group 2 > Group 4 > Group 5 > Group 3 > Group 6 > Group 1. The maximum depth of penetration in the middle level of the root was seen in Group 2, whereas the least depth of penetration in the middle level of the root was seen in Group 1.

**Table 1:** Comparison of the depth of penetration of six groups into dentinal tubules at the middle level (µm)

Group	N	Mean	SD	SE	MIN	MAX	Confidence level 95%	
							Lower bound	Upper bound
1	10	252.0813	34.97112	11.06681	225.857	321.778	182.1391	322.0235
2	10	470.2221	80.02734	25.32511	382.203	607.356	310.1674	630.2768
3	10	308.3281	28.36551	8.976426	264.092	348.121	251.5971	365.0591
4	10	405.6382	46.27746	14.64477	307.647	453.279	313.0833	498.1931
5	10	382.6633	37.02473	11.71669	305.034	425.538	308.6138	456.7128
6	10	281.6891	37.50437	11.86847	203.523	344.513	206.6804	356.6978

Depth of penetration of six groups in the apical level of the root

**Figure 4:**
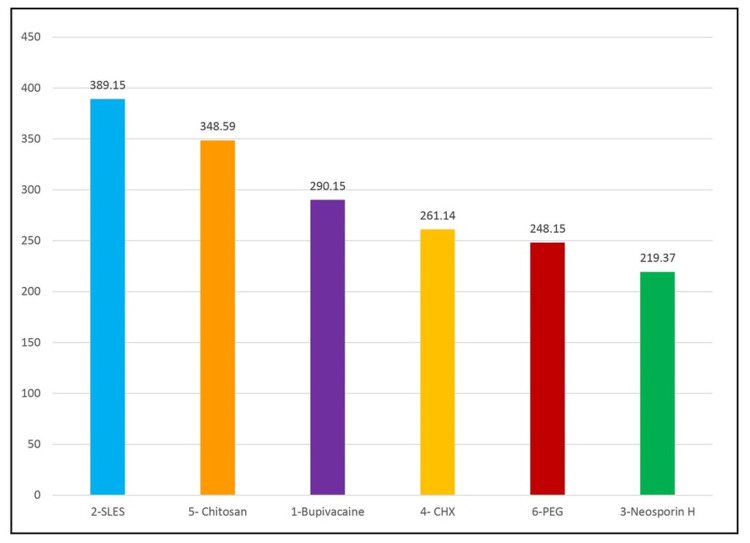
Comparison of the depth of penetration into dentinal tubules at the apical level of six groups (µm)

The mean depth of vehicle penetration in the apical level of the root for Group 1 was 290.1473 ±15.35027 µm, that for Group 2 was 389.1529 ± 8.500676 µm, that for Group 3 was 219.374± 15.45949 µm, that for Group 4 was 261.1449± 19.07977 µm, that for Group 5 was 348.5948± 13.75311 µm, and that for Group 6 was 248.1468± 7.45115 µm (Table 2). The order of depth of penetration in the apical level as seen in Figure 4 is Group 2 > Group 5 > Group 1 > Group 4 > Group 6 > Group 3. The maximum depth of penetration in the apical level of root was seen in Group 2, whereas the least depth of penetration in the apical level of root was seen in Group 3.

**Table 2:** Comparison of the depth of penetration of six groups into dentinal tubules at the Apical level (µm)

Group	N	Mean	SD	SE	MIN	MAX	Confidence Limits	
							Upper	Lower
1	10	290.1473	48.50685	15.35027	221.435	366.229	193.1336	387.161
2	10	389.1529	26.86214	8.500676	353.207	443.517	335.4286	442.8772
3	10	219.3741	48.852	15.45949	147.464	279.896	121.6701	317.0781
4	10	261.1449	60.29209	19.07977	202.694	351.354	140.5607	381.7291
5	10	348.5948	43.45982	13.75311	303.489	417.167	261.6752	435.5144
6	10	248.1468	23.54563	7.45115	216.029	286.679	201.0555	295.2381

Depth of penetration in dentinal tubules of middle and apical levels for individual groups

**Figure 5:**
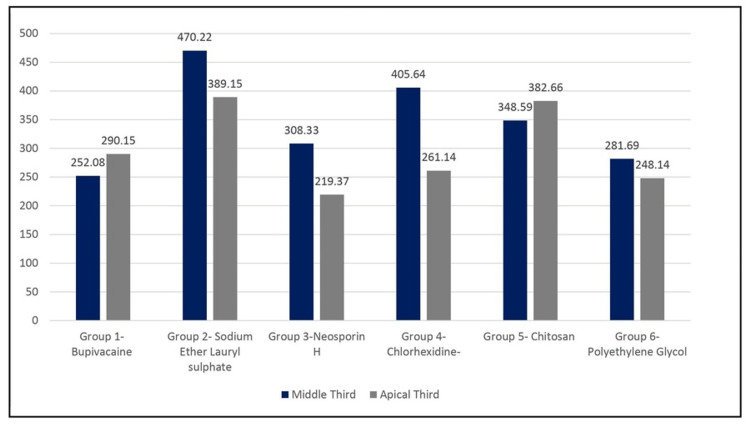
Depth of penetration in the middle and apical third of individual groups (µm)

There was no significant difference in the depth of penetration of Group 1-Bupivacaine+ MTAP, Group 5- Chitosan+ MTAP, and Group 6-Polyetheylene glycol+ MTAP at the middle and apical levels. The depth of penetration of Group 2- Sodium ether lauryl sulfate + MTAP, Group 3-Neosporin H+ MTAP, and Group 4- Chlorhexidine+ MTAP at the middle was statistically superior to the apical level (Figure 5).

Overall depth of penetration of six groups into the dentinal tubules

**Figure 6:**
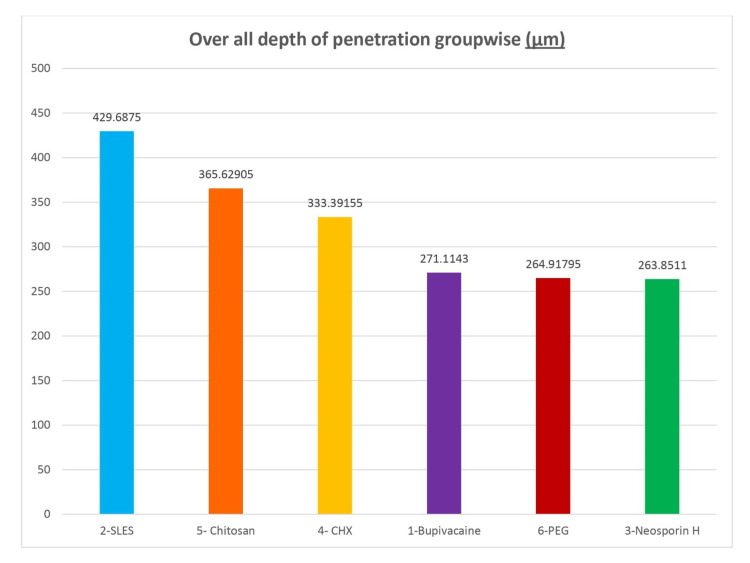
The overall depth of penetration of six groups into the dentinal tubules (µm)

The overall depth of penetration for Group 1-Bupivacaine + MTAP was 271.1143 µm, that for Group 2-Sodium Ether Lauryl Sulfate+ MTAP was 429.6875 µm, that for Group 3-Neosporin H+ MTAP was 263.8511 µm, that for Group 4- Chlorhexidine + MTAP was 333.3916 µm, that for Group 5- Chitosan + MTAP was 365.6291 µm, and that for Group 6- Polyethylene Glycol+ MTAP was 264.918 µm. The maximum depth of penetration was seen in Group 2 followed by Group 5, Group 4, Group 1, and Group 6, and the least penetration by Group 3 (Figure 6).

Depth of penetration into the dentinal tubules for the six groups according to the middle and apical levels

**Figure 7:**
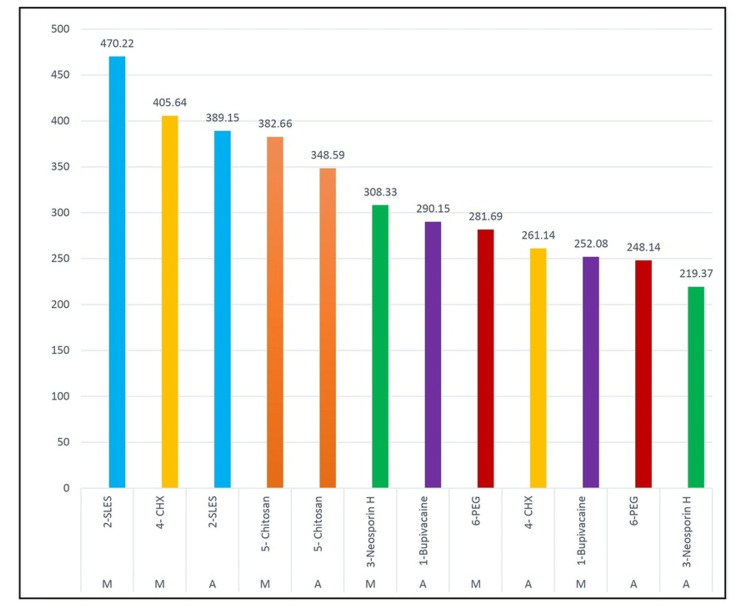
The depth of penetration into the dentinal tubules of six groups according to the middle and apical level (µm)

According to Figure 7, the order of depth of penetration into dentinal tubules is as follows: Middle level- Group 2 >Middle Level-Group 4 >Apical Level-Group 4 >Middle level Group 5 >Apical level- Group 5 > Middle Level-Group 3 >Apical Level-Group 1 >Middle level- Group 6 >Apical level- Group 4 >Middle level- Group 1 >Apical Level-Group 6 >Apical Level-Group 3.

Overall depth of penetration in dentinal tubules of the middle level and apical level

**Figure 8:**
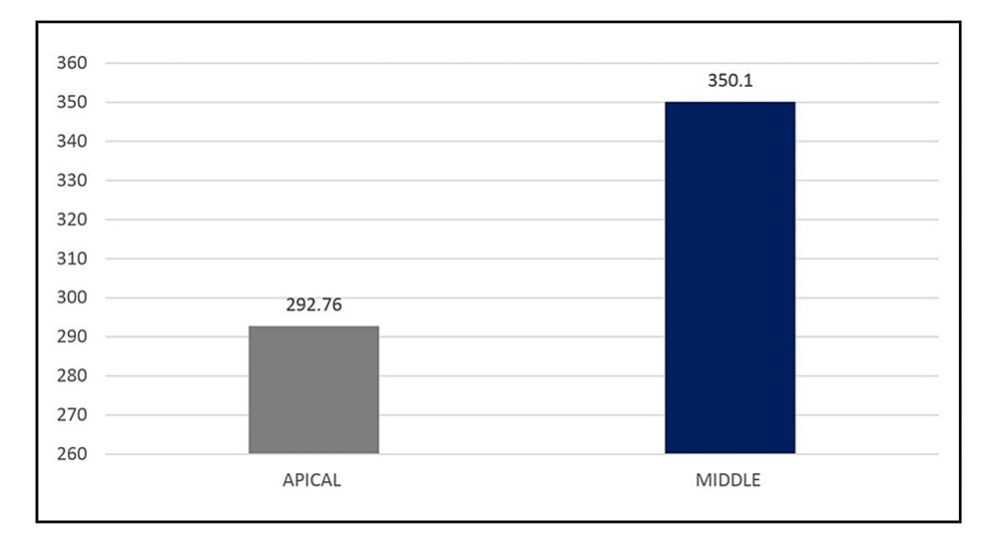
The overall depth of penetration into the dentinal tubules of middle and apical levels (µm)

The mean depth of penetration of MTAP and vehicles in the middle level was 350.1 µm and in the apical level was 292.76 µm. Hence, the overall depth of penetration was significantly higher at the middle level than at the apical level (Figure 8).

## Discussion

In the present study, single-rooted mandibular first premolars were selected to help in the standardization of the samples. The Universal Protaper system was used due to its flexibility, varying tapers, and non-cutting safety tips. The elimination of the smear layer enhances the penetration of medicament; hence, 3% Sodium hypochlorite and 17% EDTA were used [5]. The modification in MTAP of using clindamycin instead of minocycline prevented tooth discoloration. MTAP is effective against Prevotella and Porphyromonas [6]. The depth of penetration of the MTAP is of vital importance, as this helps us assess the spread of antibiotics into the root canal dentinal tubules. Among the vehicles used for the study, Bupivacine, Neosporin-H, and chlorhexidine were aqueous. Sodium ether lauryl sulfate, chitosan in acetic acid, and polyethylene glycol were the viscous vehicles.

Group 1 was bupivacaine mixed with MTAP. This is the first study using this long-acting local anesthetic drug as a vehicle for dentinal tubule. Following the fact that lower surface tension leads to better penetrability, Ozcelik et al. (2000) found that the surface tension of anesthetic solution was 44 dyne/cm, and when mixed with calcium hydroxide, it was 51 dyne/cm [7]. Compared to other vehicles in the study, i.e., glycerin, saline, and ringer lactate, the local anesthetic had the lowest surface tension.

Group 2 used sodium ether lauryl sulfate as the vehicle. The chief property is that it is a surfactant, which is a substance that tends to reduce the surface tension of a liquid in which it dissolves. This increases the wettability of the substance. Barbosa et al. (1994) stated that the addition of detergent "lauryl diethylene glycol ether sodium sulfate 0.125%" improved the efficiency of the calcium hydroxide solution by decreasing the surface tension [8].

Group 3 included Neosporin H with MTAP. Otosporin has been used in literature as a vehicle. It has been recommended due to the steroids present, which may reduce pain and associated inflammation [9]. Pecora et al. (1991) measured the surface tension of two vehicles: Otosporin (40.90 dyne/cm) and lauryl diethylene glycol ether sodium sulfate (37.52 dyne/cm) [4]. Similar outcomes were observed in this study. Estrela et al. (2005) calculated the surface tension of various vehicles and found that sodium ether lauryl sulfate had the least (31.60 dyne/cm) [10]. Sodium ether lauryl sulfate was compared with Otosporin with calcium hydroxide (35.40 dyne/cm), 2% chlorhexidine and calcium hydroxide (58.00 dyne/cm), CMCP, and sodium hypochlorite.

Group 4 included chlorhexidine with MTAP. Chlorhexidine was chosen due to its known property of substantivity. The depth of penetration is inversely related to the surface tension. In accordance with the current study, Poorni et al. (2009) observed that chlorhexidine had the lowest surface tension values in comparison with distilled water, glycerin, saline, and anesthetic solutions [11].

Group 5 included chitosan with MTAP. This vehicle has been used in a few studies because of its sustained release effect, biodegradability, and antibacterial and antifungal effects. Abiding by the fact that a lower contact angle lowers the surface tension and improves wetting of the radicular dentin, Kotadia et al. (2017) observed a lower contact area in the group of TAP and chitosan when compared to the group of calcium hydroxide and chitosan [12]. The inference was that there was better penetration of the TAP and chitosan group over calcium hydroxide and chitosan, which was in accordance with the study.

Group 6 included polyethylene glycol and MTAP. PEG-300 and PEG-400 are known vehicles for the transport of drugs through the skin. According to Carreira et al. (2007), there is a synergistic effect when PEG is used along with ciprofloxacin, which helps in eliminating yeasts as well as bacteria. Also, the addition of PEG to metronidazole and ciprofloxacin enhances dentinal tubule penetration. This fact could be useful while using PEG with MTAP [13]. The density and permeability of tubules are said to decrease from coronal to apical [14], and the irrigants are less effective in smear layer removal in the apical third [15]. This could be the reason for lesser penetration in the apical third area.

Rhodamine dye is water-soluble with a low molecular weight, which leads to penetration in the dentinal tubule. The use of a confocal laser scanning microscope provides a panoramic view of intracanal medication in dentinal tubules, its penetration at high magnifications, and serial sections even from thick specimens. The limitations of the study were that it was an in vitro study; hence, there was a possibility of differences in inferences when compared to clinical situations.

## Conclusions

For the success of root canal treatment, the elimination of microbes is imperative. Irrigation and local administration of antibiotics have shown a significant reduction in microbial loads in the canals. For local drug delivery of intracanal medicaments, various vehicles are used. In this study, among the six vehicles used, sodium ether lauryl sulfate, a known surfactant, showed the maximum depth of penetration in both the middle and apical areas, followed by chitosan and then polyethylene glycol. The exploration of various combinations of medications and vehicles for generating the ideal inter-appointment medicament continues.
